# The Recruitment of Neutrophils to the Tumor Microenvironment Is Regulated by Multiple Mediators

**DOI:** 10.3389/fimmu.2021.734188

**Published:** 2021-09-10

**Authors:** Shuvasree SenGupta, Lauren E. Hein, Carole A. Parent

**Affiliations:** ^1^Life Sciences Institute, University of Michigan, Ann Arbor, MI, United States; ^2^Department of Pharmacology, University of Michigan Medical School, Ann Arbor, MI, United States; ^3^Cancer Biology Graduate Program, University of Michigan Medical School, Ann Arbor, MI, United States; ^4^Rogel Cancer Center, University of Michigan Medical School, Ann Arbor, MI, United States; ^5^Department of Cell and Developmental Biology, University of Michigan Medical School, Ann Arbor, MI, United States

**Keywords:** neutrophils, tumor-associated neutrophils (TANs), chemokines, TGF-β, EMT, secretory pathways, secretory autophagy, EVs

## Abstract

Neutrophils sense and migrate towards chemotactic factors released at sites of infection/inflammation and contain the affected area using a variety of effector mechanisms. Aside from these established immune defense functions, neutrophils are emerging as one of the key tumor-infiltrating immune cells that influence cancer progression and metastasis. Neutrophil recruitment to the tumor microenvironment (TME) is mediated by multiple mediators including cytokines, chemokines, lipids, and growth factors that are secreted from cancer cells and cancer-associated stromal cells. However, the molecular mechanisms that underlie the expression and secretion of the different mediators from cancer cells and how neutrophils integrate these signals to reach and invade tumors remain unclear. Here, we discuss the possible role of the epithelial to mesenchymal transition (EMT) program, which is a well-established promoter of malignant potential in cancer, in regulating the expression and secretion of these key mediators. We also summarize and review our current understanding of the machineries that potentially control the secretion of the mediators from cancer cells, including the exocytic trafficking pathways, secretory autophagy, and extracellular vesicle-mediated secretion. We further reflect on possible mechanisms by which different mediators collaborate by integrating their signaling network, and particularly focus on TGF-β, a cytokine that is highly expressed in invasive tumors, and CXCR2 ligands, which are crucial neutrophil recruiting chemokines. Finally, we highlight gaps in the field and the need to expand current knowledge of the secretory machineries and cross-talks among mediators to develop novel neutrophil targeting strategies as effective therapeutic options in the treatment of cancer.

## Introduction

Neutrophils are the body’s first responders to injury or infection. They have an unparalleled ability to migrate toward gradients of chemoattractants, which are released at sites of inflammation, and to clear pathogens or cell debris by using a plethora of functions including phagocytosis, the release of cytotoxic enzymes or reactive oxygen species (ROS), and the release of neutrophil extracellular traps (NETs) (1). In addition, neutrophils have been reported in the tumor microenvironment (TME) (2), which has been described as a site of persistent inflammation similar to “*wounds that do not heal*” (3). The TME harbors a wide variety of diffusible mediators released from both tumor and stromal cells. These mediators induce neutrophil migration toward tumors and alter neutrophil function to promote or limit cancer progression. While studies have focused on understanding the tumor promoting or impairing properties of neutrophils, many questions remain unanswered about the identity of the mediators that control neutrophil recruitment to tumor sites and the function of tumor-associated neutrophils, referred to as TANs. In this perspective, we present an overview of the functions of TANs and the different classes of mediators that have been linked to neutrophil recruitment to tumors, discuss how cancer-associated changes such as the epithelial to mesenchymal transition (EMT) upregulate the expression of the mediators, and review the secretory mechanisms that potentially underlie the release of the mediators in the TME. Finally, we discuss our current understanding of the crosstalk between mediators, with a special focus on TGF-β and chemokines, to provide insights into the integrated mechanisms underlying neutrophil recruitment to the tumor niche and suggest gaps in knowledge that need to be filled for the development of anti-cancer therapeutic interventions.

## Plasticity of Neutrophils in Cancer

To phenotypically classify TANs and their wide range of functions that impact the outcomes of tumors, several categories have emerged: high-density/low-density, immature/mature, and anti-tumor/pro-tumor/pro-metastatic. Evidence is now suggesting that neutrophils exhibit phenotypic plasticity and can exist on a spectrum within any of these overlapping categories (4–7). For example, neutrophils are often described as “N1” (anti-tumor) or “N2” (pro-tumor) (8), but Zilionis et al. recently described a range of five neutrophil subsets in human lung cancer based on transcriptome analysis, with gene expression ranging from canonical neutrophil genes (N1 subset) to genes that were tumor-specific (N5 subset) (4). These seemingly different phenotypes are mostly generated due to exposure to specific mediator(s), either systemically in the bone marrow/blood or locally at the tumor sites. Studies using murine tumor models report that the immunosuppressive cytokine TGF-β is responsible for promoting the generation of neutrophils with a pro-tumoral “N2” phenotype (8), while the type I interferon IFN-β gives rise to an anti-tumoral “N1” phenotype (9). Further, the enzyme protease cathepsin c, secreted by breast cancer cells, has been reported to initiate a signaling cascade in mouse models that recruits neutrophils to the lung metastatic niche and promotes a pro-metastatic phenotype, and its secretion is correlated with shortened metastasis-free survival in humans (10).

Several neutrophil effector functions that support tumor progression have been identified, including immunosuppression, remodeling of the extracellular matrix (ECM), and promoting angiogenesis (11). For example, it has been demonstrated in mouse models that immunosuppressive neutrophils promote metastasis by releasing high levels of inducible nitric oxide synthase (iNOS), which inhibits proliferation of cytotoxic T cells (12). Additionally, the release of NETs from neutrophils, which are mesh-like structures of DNA fibers studded with granule proteins, is associated with metastatic progression (13–16). In particular, granule proteins such as neutrophil elastase (NE) and matrix metalloproteinase 9 (MMP9) released with NETs have been shown to awaken dormant cancer cells by remodeling the ECM and inducing cancer cell proliferation in mouse models (17).

Although most studies have reported a pro-tumor effect of neutrophils, some contexts exist where neutrophils exhibit anti-tumor effects, seemingly maintaining their canonical role to protect the body from harm. These anti-tumor actions of neutrophils in mouse models include the direct killing of cancer cells through the release of cytotoxic ROS (including hydrogen peroxide) and the restriction of tumor growth by stimulating cancer cell detachment from the basement membrane by the release of MMP9 (18–20). Additionally, the neutrophil-dependent stimulation of T cell responses can indirectly contribute to anti-tumor activity, shown both in mouse models and in cells isolated from human tissue samples (11, 21). While it has been reported that specific mediators contribute to the pro- or anti-tumoral actions of neutrophils, the mechanisms underlying how the mediators are expressed and secreted by cancer cells and the interplay among the different mediators in regulating neutrophil function remain unknown.

## Diffusible Mediators in the Tumor Niche

Cancer cells and stromal cells, including cancer-associated fibroblasts (CAFs), T cells, monocytes, tumor-associated macrophages, and TANs, can secrete diverse mediators that diffuse through tissues, potentially signaling to circulating or tissue-patrolling neutrophils to recruit them to the tumor niche. **Table 1** provides a list of recently identified neutrophil mediators released in various mouse and human tissue or cell line models of cancer and their sources. These mediators primarily consist of chemokines, growth factors, and cytokines. The chemokines CXCL1, 2, 5, 6 and 8, which induce neutrophil chemotaxis through CXCR2/1 chemokine receptors, have been reported to be important for neutrophil recruitment in many cancer types (22, 25–28, 30–38). Chemokines are critical to recruit neutrophils not only to primary tumor sites but also to pre-metastatic niches and metastatic sites. For example, CXCL1, 2, and 5 released from tumor-associated mesenchymal stromal cells in a mouse model of breast cancer resulted in increased neutrophil recruitment to primary tumor sites (22). Additionally, CXCL5 and 7 released from tumor-activated platelets were reported to be crucial for neutrophil recruitment to the pre-metastatic niche and subsequent tumor cell seeding in the mouse lung (26). G-CSF and GM-CSF are important growth factors commonly upregulated in cancer, and their primary functions are to regulate the release of mature neutrophils from the bone marrow into the blood and to extend the survival of neutrophils (38, 39). In addition, cytokines, including interleukins (IL-17A, IL-6) (10, 12, 25, 40), interferons (IFN-β) (9), TNF-α (25), and TGF-β (5, 8, 24, 28), have been linked to neutrophil recruitment and extended neutrophil survival, as well as regulating neutrophil function. While a growing number of studies report the effect of individual mediators on neutrophil recruitment and function, it is likely that antagonistic, additive, or synergistic effects of different classes of mediators are crucial for neutrophil recruitment and function in the context of cancer.

**Table 1:** List of diffusible mediators along with their cellular origin, potential impact on neutrophils and tumor progression, and the study models.

Cancer Type	Source of diffusible mediators in the TME	Diffusible Mediators	Potential impact on neutrophils	Model	Ref
breast cancer	tumor-associated mesenchymal stromal cells	CXCL1, CXCL2, CXCL5	increased migration to tumor site	mouse	(22)
	γδ T cells at tumor site	IL-17	increased migration to tumor site, change to pro-tumor phenotype, increased metastasis	mouse	(12)
	origin unclear	G-CSF (induced by IL-17)			
	cancer cells	G-CSF	recruitment to metastatic sites	mouse	(23)
	cancer cells	chemokines active *via* CXCR2, TGF-β	increased migration	human (C)	(24)
	cancer cells	IL-6, CCL3 (induced by cathepsin c)	increased migration to tumor site, NET formation, ROS production; pro-metastasis (cathepsin c works *via* the PR3-IL-1β-NF-κB axis of neutrophils to upregulate secretion of IL-6 and CCL3)	mouse	(10)
colorectal cancer	γδ T cells at tumor site	CXCL8 (IL-8), GM-CSF	increased migration	human (T)	(25)
		IL-17A, GM-CSF	expansion of the PMN-MDSC population		
		CXCL8 (IL-8), IL-17A, TNFα	extended survival		
	platelets interacting with tumor cells in pre-metastatic niche	CXCL5, CXCL7	increased recruitment to early pre-metastatic niche	mouse	(26)
	cancer cells	CXCL1	increased recruitment to tumor site and tumor progression	mouse	(27)
	cancer cells	CXCL5	increased recruitment to tumor site and increased metastasis	mouse	(28)
	origin unclear	TGF-β	increased recruitment to metastatic site		
	Th17 cells	CXCL8 (IL-8)	increased migration	human (T)	(29)
hepato-cellular carcinoma	tumor-associated monocytes	CXCL2, CXCL8	increased migration and extended survival	human (T)	(30)
	cancer cells	CXCL5	increased migration	mouse; human (C)	(31, 32)
lung cancer	tumor-associated monocytes, macrophages, neutrophils, and DCs	CXCL1	these migration-inducing chemokines are shown to have elevated levels of mRNA	mouse	(33)
	TANs	CXCL2			
	cancer cells	CXCL5			
melanoma	TANs	CXCL1, CXCL2	increased migration to tumor site and angiogenesis	mouse	(34)
ovarian cancer	tumor cells	CXCL8 (most impact) and other chemokines active *via* CXCR2	increased migration	human (T)	(35)
pancreatic cancer	cancer cells	CXCL5	increased migration to tumor site *via* CXCR2	mouse	(36)
	stromal cells	CXCL2			
	PDAC tumors	GM-CSF, G-CSF	these migration-inducing chemokines are shown to have elevated levels of mRNA	human (T)	(37)
	neoplastic ductal cells	CXCL1, CXCL2, CXCL5, CXCL8		human (T)	
	tumor cells	GM-CSF, G-CSF, CXCL1, CXCL2, CXCL5	increased migration to tumor site *via* CXCR2	mouse	
thyroid cancer	cancer cells	chemokines active *via* CXCR1/2	increased migration	human (C)	(38)
		GM-CSF	increased survival		

The model type is specific for the experiments that detailed the impact on neutrophils. C, cell line; T, tissue.

## Mechanisms Regulating the Secretion of Diffusible Mediators From Cancer Cells

**Figure 1:**
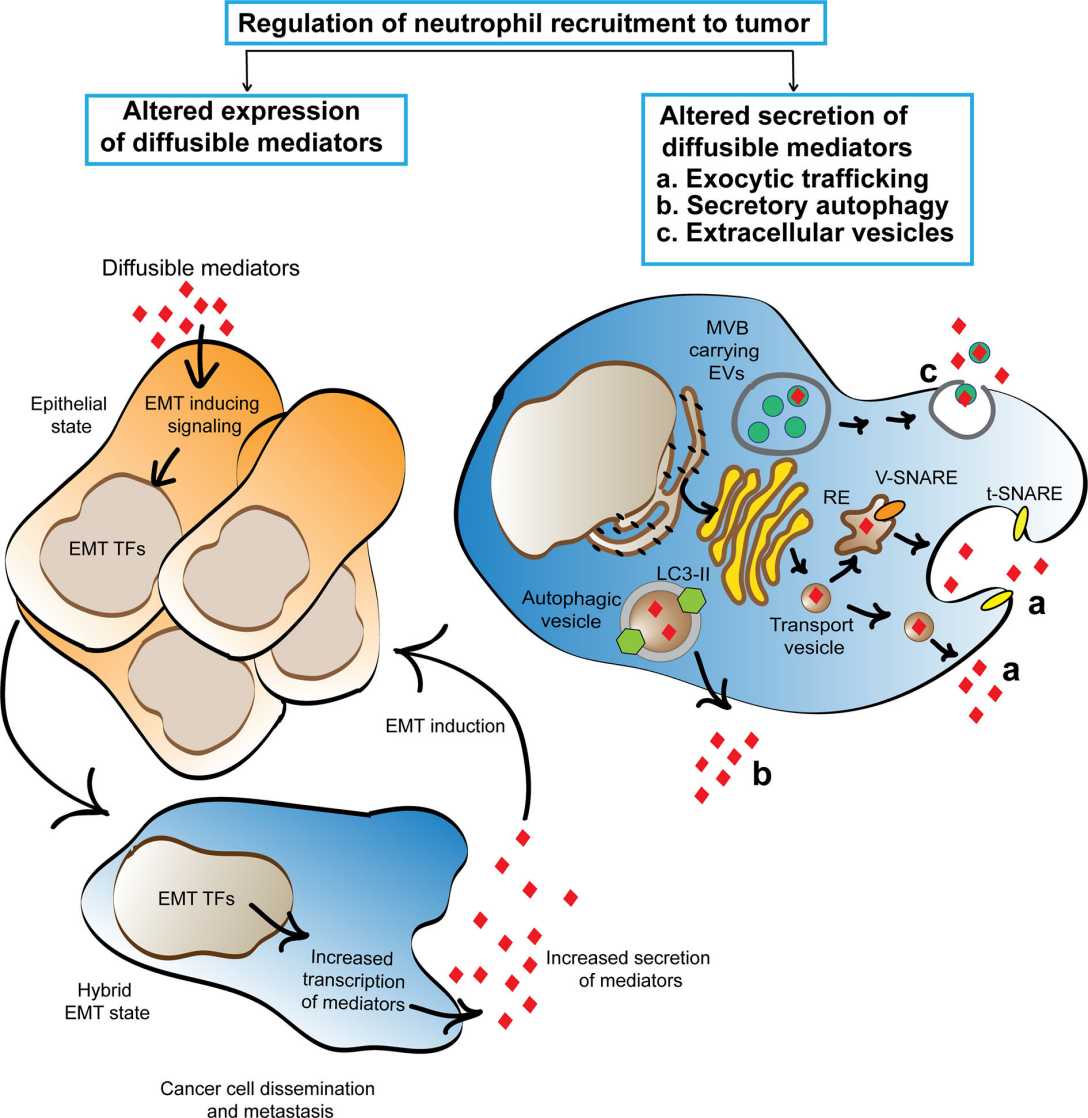
Cartoon depicting the proposed mechanisms that regulate the cancer secretome and favor neutrophil trafficking to tumors. Cancer associated EMT program activation alters cancer cell secretome by inducing the expression of neutrophil recruiting mediators. Three machineries, namely exocytic trafficking, secretory autophagy, and extracellular vesicles are proposed to enhance the release of neutrophil recruiting mediators from cancer cells. The secreted mediators promote EMT using a feed forward mechanism and initiate a chronic cycle of inflammation that supports cancer cell dissemination. TFs, transcription factors; RE, recycling endosome.

Cancer cells are known to upregulate the transcription of many diffusible mediators due to the constitutive activation or overexpression of oncoproteins (41). In many cases, the higher expression of the mediators correlates with poor clinical progression (42, 43). However, little is known about the secretory mechanisms that regulate the release of the mediators from cancer cells into the tumor niche and how the process of secretion may be altered due to cancer-associated changes compared to the mechanisms observed in non/early malignant cells. Here, we suggest that EMT induction is a key process that alters the tumor secretome and highlight the mechanisms and molecular players known to regulate the secretion of the mediators. We envision that similar mechanisms underlie the secretion of neutrophil recruiting mediators from cancer cells (**Figure 1**).

### Epithelial to Mesenchymal Transition

EMT is classified into three subtypes based on the biological context. Type I EMT is associated with embryonic development and multiple organ formation. Type 2 EMT is involved in wound healing through tissue repair and regeneration, which if unrestrained, could lead to tissue fibrosis, and organ damage. Type 3 EMT is exclusively associated with malignancy and metastatic spread, where cancer cells acquire the ability to invade locally and disseminate systemically (44). Epithelial cells undergoing all three types of EMT tend to lose their epithelial characteristics and acquire migratory mesenchymal cell-like properties. However, EMT is emerging as a dynamic process where cells adopt partial EMT or intermediate/hybrid states featuring a combination of phenotypes of both cell types (45–47). Type 3 EMT (subsequently referred to as EMT) triggers cytoskeletal remodeling, loss of cell-cell adhesion and cell polarity, and gain of migratory and invasive properties, which are proposed to be required for metastasis. A wide range of diffusible mediators released from transformed or non-transformed cells in the tumor niche are known to induce EMT, including growth factors (EGF), cytokines (TGF-β, TNF-α), chemokines (CXCL8, CXCL6), and lipid mediators (leukotriene B_4_ (LTB_4_)) (48, 49). Interestingly, EMT has been associated with altered secretory profiles of cancer cells (50–54). For instance, secreted factors from EMT-positive breast cancer cell lines have been reported to induce the *in vivo* recruitment of granulocytic myeloid derived suppressor cells (G-MDSC), which phenotypically resemble murine neutrophils and share immunosuppressive functions with “N2” neutrophils (50, 55). EMT-induced altered secretome of breast cancer cell lines also favors a tumor-permissive niche by activating tumor-associated macrophages, which further support EMT induction of cancer cells (54).

EMT inducing signals mediate their effects by stimulating a transcription program *via* the activation or enhanced expression of key EMT transcription factors: SNAIL, Twist and Zeb family proteins, and the T-box transcription factor Brachyury (56, 57). The role of these transcription factors in suppressing epithelial cell-cell adhesion proteins and inducing mesenchymal adhesion molecules has been widely studied (58, 59). Interestingly, the same transcription factors are also emerging as key regulators for the expression of mediators such as cytokines (TNF-α), chemokines (CCL2, CXCL6, GRO, CXCL8, CXCL11), and growth factors (GM-CSF) in cancer cells (50, 52, 53, 60–63). For example, chemokines, including CXCL6 and CXCL8, are one of the many secreted mediators that are upregulated in a Snail-dependent manner when EMT pathways are activated in cancer cells by EGF or TGF-β treatment (50). As many of the secreted mediators from EMT-activated tumors are established chemoattractants of neutrophils (GRO, CXCL8, GM-CSF) and monocytes (CCL2), the release of these mediators upon EMT induction is poised to regulate the immune landscape of the tumor niche.

#### Exocytic Trafficking Pathways

Conventional mechanisms that underlie the secretion of diffusible mediators, such as cytokines and chemokines, involve constitutive and regulated exocytosis pathways, depending on the cellular and inflammatory context (64–66). Much of our current knowledge comes from characterization in immune cells, particularly macrophages and dendritic cells (DCs) for constitutive secretion, and granulocytes for regulated secretion. In general, cytokines and chemokines carry a leader peptide sequence for secretion that facilitates their trafficking through the ER-Golgi network. Newly synthesized proteins are continuously exocytosed through trafficking from the Golgi network to the plasma membrane *via* small transport vesicles or tubules, which transport the cargo to the plasma membrane either directly or by merging with recycling endosomes (67). In contrast, pre-formed proteins after transiting through the Golgi network are stockpiled in vesicles or granules, which undergo regulated exocytosis in response to external inputs through receptor-ligand interactions (65). Key molecular players of the trafficking machinery include the evolutionary conserved membrane fusion proteins of the soluble N-ethylmaleimide-sensitive factor (NSF) attachment protein receptor (SNARE) protein family (67). Fusion of the vesicle and target membrane to form the core-SNARE complex is mediated by V (vesicle-associated)-SNARE and t (target membrane)-SNARE members for both constitutive and regulated exocytosis. For instance, the V-SNARE member vesicle-associated membrane protein 3 (VAMP3) localized in recycling endosomes mediates the membrane fusion of CXCL6- and TNF-α-carrying vesicles with the plasma membrane that leads to their constitutive secretion from macrophages and DCs (68–70). In granulocytes, such as eosinophils, however, CXCL6 is released through receptor mediated degranulation or regulated exocytosis, where the function of late endosomal V-SNARE members such as VAMP2 and VAMP7 have been implicated (71–74). VAMP7 is also reported to control the release of CXCL12 from VAMP7-positive late endosomal compartments in DCs, suggesting that the trafficking machinery varies greatly depending on cell types and mediators. Further, both V-SNAREs and t-SNAREs may play a rate-limiting role as their upregulation has been noted in stimulated macrophages and DCs with concomitant increase in mediator secretion (70, 75, 76). In addition, other molecular players such as Rho GTPases, including Rac1 and Cdc42, play important roles in TNF secretion in macrophage by delivering TNF-carrying recycling endosomes to the cell surface (77).

While cytokine trafficking in epithelial cells most likely utilizes the constitutive pathway (64), the mechanisms underlying mediator secretion are not well established in malignant cells even though cancer cells are known to abundantly secrete diverse mediators. An upregulation of signature genes associated with ER to Golgi trafficking pathways has been linked to the increased secretion of mediators, including CCL20, from murine breast cancer cell lines with high metastatic potential (78). Moreover, it has been shown that the secretion of CCL5 depends on the exocytosis of CCL5-carrying pre-made vesicles in the hormone receptor positive breast cancer cell line MCF-7 (79). Whether specific VAMP proteins mediate CCL5 trafficking and vesicle fusion with the plasma membrane and if/how the machinery differs in early- and late-stage malignant cells compared to non-malignant epithelial cells have yet to be determined. Of note, VAMP3 has been reported to be involved in CXCL6 and TNF-α release from the synovial sarcoma cell line SW982, indicating an active role of VAMP proteins in diffusible mediator secretion from cancer cells (80). Furthermore, studies have reported enhanced expression of V-SNARE and t-SNARE members in cancer cells along the course of tumor progression, which may further promote the exocytic release of the mediators in the TME (81).

#### Autophagy

An unconventional mechanism for diffusible mediator secretion is autophagy. Autophagy is traditionally known for intracellular degradation and recycling of cargos, including damaged organelles or protein aggregates, to maintain cellular homeostasis. The fusion of autophagic vacuole carrying cargos with lysosomes results in cargo degradation by acid hydrolysis and proteolysis (82). Our understanding of the machinery involved in autophagy-dependent secretion, also known as secretory autophagy (SA) (83), is however less clear. Under nutrient deprived conditions in yeast, SA is known to mediate the release of leader peptide-less proteins (84). However, there is emerging evidence that SA is involved in the secretion of mediators that are both leader-less, such as IL-1β and IL18, and leader-positive, such as CXCL6, CXCL8, and TGF-β (85, 86). Attempts to define the sequence of events leading to SA of IL-1β from macrophages in response to lysosomal damage identified several molecular players key to the process (87). For example, TRIM16 serves as the SA cargo receptor that together with VAMP member Sec22b sequesters IL-1β in LC3-II-positive vesicles, where LC3-II is a canonical autophagosome marker. Further, the fusion of cargo vesicles with the plasma membrane as well as the release of cargos are achieved by the coordinated action of dedicated SNARE proteins including syntaxin 3, syntaxin 4, SNAP-23, and SNAP-29.

SA in both cancer cells and CAFs has recently been implicated in shaping the tumor secretome and promoting cancer progression (88, 89). For instance, secretion of CXCL8, IL-1β, LIF (leukemia inhibitory factor), and Fam3 (family with sequence similarity 3 member C) were found elevated or impaired in a murine melanoma cell line when stimulated with an autophagy-inducing peptide or subjected to autophagy related gene knockdown approaches, respectively (90). More strikingly, a correlation between the elevated presence of the same mediators in the serum of patients with high-autophagy melanoma, compared to patients with low-autophagy melanoma, was reported in the same study. Autophagy is also recognized to contribute to oncogenic RAS driven cancer cell migration and invasion by inducing the secretion of the migration promoting CXCL6 chemokine and the transcription of pro-invasive molecules, including MMP2 and WNT5A (91). In addition, CXCL6 secretion was mediated by autophagy in the triple-negative breast cancer cell line MDA-MB-468, which depends on autophagy for survival (92). In contrast, autophagy inhibition promoted CXCL6 secretion from MCF-7 cells, which otherwise does not depend on autophagy for survival. The apparent contrasting effect of autophagy on CXCL6 secretion reflects context dependent regulation of cytokine secretion by autophagy and highlights the need to explore more comprehensively the role of autophagy in mediator secretion in different cancer types and subtypes.

#### Extracellular Vesicles

Extracellular vesicles (EVs) are heterogeneous in size (93). Exosomes are smaller EVs with a diameter less than 150 nm that originate as intraluminal vesicles (ILVs) by the inward budding of late endosomal vesicles that form multivesicular bodies (MVBs). Upon fusion of MVBs with the plasma membrane, ILVs are released as exosomes in the extracellular milieu. By contrast, other larger EVs (diameter up to 1000 nm) are generated through the outward budding of the plasma membrane (93) and come in different flavors, including microvesicles or ectosomes, migrasomes (secreted along retraction fibers of migrating cells), and oncosomes (secreted by cancer cells) (93–96). EVs are well established as vehicles for diverse cargos, including proteins, lipids, and nucleic acids that mediate intercellular communication. EVs released from cancer cells, CAFs, and immune cells have been shown to induce directional migration of the same or other cell types through autocrine and paracrine communication (93, 97). Interestingly, cancer cell-secreted exosomes were shown to mediate the systemic mobilization of neutrophils to the spleen in an *in vivo* model of breast cancer (98). However, the role of exosomes as the vehicle for tumor-secreted mediators that directly induce neutrophil migration remains to be determined. A diverse group of cytokines, chemokines and growth factors were found to be associated at the surface of EVs or encapsulated inside EVs that were isolated from cultured immune cells, tissue explants, and different types of biological fluids (99). The availability of the mediators in a free or EV-associated form was reported to depend on the activating stimuli and the cellular system studied. Furthermore, CCL chemokines were found to be enriched in exosomes when tumor cells were exposed to heat stress (100). The degree of exosomal chemokine release may therefore be tunable as tumor cells are exposed to changing physicochemical factors in the dynamic TME. EVs are also emerging as a vital means of tumor-stromal cell communication that further promote tumor progression and metastasis. For instance, osteosarcoma cells release EVs carrying membrane-associated TGF-β1, which was shown to educate mesenchymal stromal cells to release CXCL6, and promote further tumor growth and metastasis (101). In addition, osteosarcoma-derived EVs were shown to induce lung fibroblast differentiation in a TGF-β1 dependent manner, indicating a potential role of EV-associated immune mediators to endorse distant metastasis (102).

## Interplay Between TGF-β and Chemokines to Maximize Neutrophil Recruitment

As mentioned, given the presence of diverse cell types in the tumor niche, the TME harbors multiple diffusible mediators. Neutrophil navigation to the tumor niche could therefore be orchestrated by the interplay of different mediators. We recently reported that CXCR2 ligands, potentially growth-related oncogene (GRO) members (CXCL1/2/3), and TGF-β1, which are abundantly secreted by triple-negative breast cancer cells, concertedly induce robust neutrophil migration (24). TGF-β ligands belong to the TGF-β subfamily, with three known mammalian isoforms: TGF- β1, TGF-β2 and TGF-β3, of which TGF-β1 is the most commonly expressed. Cells secrete all isoforms as a latent complex that is activated by the presence of integrins, ECM proteins, and proteolytic enzymes (103). Once released in an active form, all isoforms interact and activate the type II/type I TGF-β receptor complex and propagate signals through SMAD-dependent and -independent pathways (104, 105). TGF-β target genes are involved in regulating fundamental cellular functions such as proliferation, differentiation, migration, senescence, apoptosis, along with maintaining immune homeostasis. The signaling outcome of TGF-β is highly context dependent in cancer. During early stages of cancer, it can prevent tumorigenesis by inhibiting cell proliferation, regulating cell cycle progression, and promoting apoptosis. However, cancer-associated disruption of TGF-β receptor/signaling components and/or the activation of EMT inducing signaling of TGF-β may promote the dissemination of cancer cells (105, 106). The mechanistic basis for the complexity of TGF-β signaling outcome has achieved significant clarity over the years. However, the mechanism and outcome of the crosstalk of TGF-β with other diffusible mediators on tumor progression are only beginning to be understood.

Both cancer and immune cells express TGF-β receptors. Receptor expression on immune cells is further modulated by the mediators present in the tumor niche. For instance, mediators secreted from metastatic prostate cancer cells upregulate the gene expression of the type I TGF-β receptor (TGF-β RI) in neutrophils (107), suggesting that the effect of TGF-β on neutrophil function is tunable. In an *in vivo* murine model of lung cancer and mesothelioma, TGF-β has been reported to promote the tumor supporting functions of neutrophils and treatment with a systemic inhibitor of TGF-β RI led to increased neutrophil recruitment to tumors indicating a negative regulation of neutrophil migration by TGF-β signaling (8). Conversely, TGF-β signaling was reported to promote neutrophil recruitment to metastatic sites in a genetically engineered *in vivo* mouse model of metastatic colorectal cancer (28). Additionally, *in vitro* studies documented various ways by which TGF-β can foster or hinder other neutrophil responses, such as prolonging neutrophil survival, promoting phagocytosis and respiratory burst (108), and impairing granule exocytosis (109).

The role of TGF-β in directional migration of neutrophils is, surprisingly, not clear. Studies have reported strong to no direct effect of TGF-β on neutrophil chemotaxis (110, 111). Given its pleiotropic role, TGF-β may indirectly regulate neutrophil chemotaxis. Indeed, TGF-β1 was reported to promote chemotaxis of immature DCs to CC and CXC chemokines by upregulating chemokine receptor expression (112). Whether TGF-β1 uses a similar mechanism to regulate CXCR1/2 expression and mediate its effect on neutrophil chemotaxis remains unknown. TGF-β1 has also been reported to promote the secretion of CXCL5 from hepatocellular carcinoma cell lines, which in turn induces neutrophil migration (32). Furthermore, TGF-β1 is known to enhance the secretion of leukotrienes from monocyte-derived macrophages and DCs, of which LTB_4_ is a potent neutrophil recruiting lipid mediator (113, 114). Finally, chemokines may also synergize with TGF-β to optimize cellular responses by triggering the activation of downstream signaling components, such as SMAD3, which was reported to be phosphorylated by chemokines like CCL2 (115).

## Targeting Strategies/Perspective

Our knowledge of the multifaceted functions of neutrophils in cancer is rapidly expanding. Yet, a precise understanding of the diffusible mediators that are secreted in the TME and induce neutrophil trafficking to the tumor niche is lacking. Many cancer therapeutic strategies, such as chemotherapy, radiotherapy, and immune-checkpoint inhibitors, have the potential to affect the level of circulating neutrophils or modulate the recruitment or function of TANs, which may in turn impact patient prognosis (116). More effort should therefore be placed on directly targeting the diffusible mediators themselves or the pathways that underlie neutrophil recruitment to the TME. Integrating such neutrophil-focused approaches with routinely applied therapeutic strategies may lead to a synergistic protection against cancer progression. However, the fact that neutrophils are quintessential soldiers of the immune system requires careful consideration in developing neutrophil targeting strategies for cancer therapy. From a mechanistic standpoint, it is therefore crucial to address several questions in the context of neutrophil recruitment to specifically target the process without compromising the overall protective role of neutrophils. For example, (i) which trafficking molecules regulate the secretion of neutrophil recruiting mediators in cancer cells? (ii) Does EMT induction change the expression of these regulators and enhance the release of the mediators? (iii) Does the exocytic pathway/SA/EV-dependent release of mediators further promote EMT by triggering an autocrine-paracrine loop? and (iv) How do mediators from distinct classes such as chemokines and cytokines/growth factors/lipid mediators collaborate to optimize neutrophil recruitment to tumors and reprogram TAN function? Addressing these basic questions will provide a deeper understanding of the molecular players and signaling components that dictate neutrophil trafficking to tumors, which will assist in the design of effective therapeutic strategies.

## Author Contributions

SS, LEH, and CAP equally contributed to conceptualizing, writing, and editing the manuscript. All authors contributed to the article and approved the submitted version.

## Funding

This work was supported by funding from the University of Michigan School of Medicine.

## Conflict of Interest

The authors declare that the research was conducted in the absence of any commercial or financial relationships that could be construed as a potential conflict of interest.

## Publisher’s Note

All claims expressed in this article are solely those of the authors and do not necessarily represent those of their affiliated organizations, or those of the publisher, the editors and the reviewers. Any product that may be evaluated in this article, or claim that may be made by its manufacturer, is not guaranteed or endorsed by the publisher.
